# Predicting the Mesiodistal Crown Dimensions of the Permanent First Molars from the Deciduous Second Molars

**DOI:** 10.1155/2021/9315553

**Published:** 2021-06-16

**Authors:** Dunia Ahmed Al-Dulaimy, Mohammed Nahidh, Mohammed Rafid A. Al-Khannaq

**Affiliations:** ^1^Department of POP, College of Dentistry, Mustansiriyah University, Baghdad, Iraq; ^2^Department of Orthodontics, College of Dentistry, University of Baghdad, Baghdad, Iraq

## Abstract

**Background:**

This study aims to formulate regression equations that predict the mesiodistal crown widths of the permanent first molars utilizing the mesiodistal crown widths of the deciduous second molars.

**Methods:**

Fifty pairs of study models belonging to 50 Iraqi children aged eight to nine years with sound mixed dentition were used to measure the mesiodistal crown widths of the permanent first molars and deciduous second molars using a pointed digital sliding caliper with 0.01 mm sensitivity. Side and gender differences were assessed, and the correlations between these teeth were obtained to develop the regression equations.

**Results:**

The results revealed no significant side differences, so the samples were merged and analyzed for gender differences, which were found to be significant in all examined teeth except the mandibular permanent first molar. Direct, moderate, and highly significant correlations between the mesiodistal crown widths of the permanent first molars and deciduous second molars were found, which led to the development of regression equations. After applying these equations, the resultant predicted widths were compared to the actual widths, and the results revealed nonsignificant method differences.

**Conclusions:**

A new method was developed to predict the widths of permanent first molars from the adjacent primary second molars with high precision.

## 1. Introduction

The primary teeth play important roles in esthetics, phonetics, mastication, alveolar growth, and guiding occlusion to its optimal state [1]. In order to achieve normal occlusion in adulthood, evaluation of the primary dentition is essential to recognize and solve occlusal problems in any stage of dental development [2].

One factor that affects the alignment of teeth in the dental arches and contributes to the development of occlusal relationships during the period of transition from deciduous to permanent dentition is the mesiodistal crown diameter of teeth. Mesiodistal widths of deciduous teeth and occlusion in primary dentition are crucial to the preservation of space and occlusion in permanent dentition [3].

As the primary teeth are replaced by the permanent teeth in development, changes in occlusal relation occur due to many factors. One such factor is the loss of Leeway space due to early loss of primary molars. Another factor may be the size variation in primary versus permanent teeth. Tooth size is largely dependent upon heredity. However, hereditary factors that determine the size of the teeth and the size of arches have no connection with one another. Therefore, the difference between the size of the teeth and arches can lead to crowding or spacing [4].

For the teeth to have an appropriate alignment in the dental arches, the total mesiodistal width of all the teeth should be proportional to the dental arch associated with it. The tooth size ratio represents a good diagnostic tool that can be used for scientific prediction of the outcome of any treatment and may also lessen the need for further diagnostic setups in complex cases. The most commonly studied crown dimension is the mesiodistal diameter [5].

During the mixed dentition period, the most important deciding factor in the management of occlusal development in growing children is the prediction of the size of unerupted teeth if a high-quality treatment plan is to be implemented [6–8].

Various methods have been developed to predict the width of unerupted teeth. The most frequently used methods are Moyers' probability tables [9] and the prediction equation of Tanaka and Johnston [10]. However, these methods were developed using Caucasian populations, and their predictive precision on other races is suspected. Accordingly, prediction equations and probability tables were developed for different populations [11, 12].

The permanent first molar usually erupts in the oral cavity at the age of six years and is considered the largest tooth in the oral cavity with the greatest biomechanical importance [13]. It is also thought to be the most dimorphic tooth of the permanent dentition [14].

On the other hand, the deciduous second molar has the largest mesiodistal diameter of the deciduous dentition and serves as an exact replica of the permanent first molar, though smaller in size. The morphological concordance between these teeth is known as isomorphism, and the deciduous second molar can be used as a guide for predicting the size of the permanent first molar in the same quadrant [13].

Previous studies have shown a significant correlation between the widths of the deciduous second molars and the permanent first molars. Such measurements are important for space analysis and orthodontic treatment planning. For example, this finding may be used as a prognostic for tooth-jaw disharmony that could indicate probable crowding in the permanent dentition [15–18]. The aim of this study is to develop a regression equation that can be used to predict the mesiodistal width of the permanent first molars using the mesiodistal widths of the deciduous second molars in a sample of Iraqi populations.

## 2. Materials and Methods

This retrospective cross-sectional study was approved by the scientific and ethical committee in the college of dentistry with the reference number 12-2019. The parents signed a consent form regarding participation of their children in this study.

Fifty pairs of study models were taken from 50 Iraqi children (25 males and 25 females) aged eight to nine years. The children were in the mixed dentition stage and belonged to various socioeconomic statuses. They were selected from multiple elementary schools in Baghdad city according to the following criteria:

1. The dentition is sound with no caries, proximal restorations, or loss of dental material for any reason
2. The first permanent molar and second deciduous molars are free from any abnormalities in size, shape, or structure
3. The maxillary and mandibular models do not contain voids, cracks, fractures, irregularities, or any technical defects

An electronic digital vernier caliper (Insize Co., USA) calibrated to the nearest 0.01 mm was used to measure the mesiodistal dimensions of the deciduous second molars and permanent first molars at the mesial and distal contact points parallel to the occlusal surface [3].

### 2.1. Statistical Analysis

The collected data were analyzed using SPSS software version 24 (IBM, USA). Inter- and intraexaminer reliabilities for all measurements were tested by the intraclass correlation coefficient. The means and standard deviations of the studied teeth were obtained. Side and method differences were analyzed using a paired sample *t*-test, while gender difference was analyzed using an independent sample *t*-test. Pearson's correlation coefficient test was used to study the relationships between the examined teeth; then, simple regression equations were developed based on Pearson's correlation coefficient test.

## 3. Results

All variables were remeasured by the same researcher (D.A.) after one month to test the intraexaminer reliability and by another researcher (M.R) to test the interexaminer reliability. An intraclass correlation coefficient was used and indicated excellent reliabilities (0.93 for intra- and 0.9 for interexaminer reliability).

The means and standard deviations of the mesiodistal widths of the maxillary and mandibular permanent first molars and deciduous second molars, as well as their side differences, are presented in Table 1. The results of analysis revealed a nonsignificant side difference for all teeth in both genders, so the right and left measurements were collected as one sample and tested for gender difference, as shown in Table 2. All teeth demonstrated a highly significant gender difference, except the mandibular permanent first molar.

The relationships between the mesiodistal widths of the permanent first molars and deciduous second molars for both arches and genders are presented in Table 3. In general, the correlation was found to be direct, moderate, and highly significant. Thus, an attempt was made to develop regression equations that can predict the mesiodistal widths of the maxillary and mandibular permanent first molars from the deciduous second molars (Table 3).

After applying the regression equations, the predicted mesiodistal widths of the maxillary and mandibular permanent first molars were compared to the actual widths, and the results revealed a nonsignificant difference (Table 4).

## 4. Discussion

Mesiodistal teeth width can be used in a variety of analyses and interpretations, such as the study of the changes and evolution of teeth sizes along different age groups, [5] ethnic groups [19], and various environmental conditions, in addition to their utility in predicting changes in dental arches and anticipating crowding and spacing at an early age [20]. Moreover, mesiodistal teeth width measurements can be used in forensic dentistry to detect the gender of victims [21].

Previous studies [15–18] have evidenced the presence of a direct relationship between the mesiodistal widths of the maxillary and mandibular permanent first molars and deciduous second molars. The current study was conducted in order to utilize the mesiodistal widths of the deciduous second molars to predict the mesiodistal widths of permanent first molars.

This study included fifty study models belonging to 50 males and females selected from a population of primary school children in Baghdad city, and all were aged eight to nine years and displayed caries-free teeth. Although the sample size might appear small, it was so difficult to find a sound teeth in mixed dentition at that age as primary molars are mostly affected by carious lesions in the proximal sides easily in the absence of good oral hygiene or poorly motivated child. The mesiodistal widths of their maxillary and mandibular permanent first molars and deciduous second molars were measured at the mesial and distal contact points according to the method of Moorrees et al. [3] using a fine-tipped digital sliding vernier.

The results in Table 1 revealed no significant side differences for all teeth measured. This is in accordance with other findings [15–18, 22–24]; therefore, the right and left samples were merged.

Gender difference was tested using an independent samples *t*-test, and the results showed that the males had significantly larger mesiodistal crown dimensions for all teeth except the mandibular permanent first molar, which demonstrated a nonsignificant gender difference (Table 2). This too aligns with the findings of many other studies [16, 22, 23].

Previous studies [15–17] found a strong-to-moderate direct and highly significant correlation between the crown mesiodistal widths of the maxillary and mandibular permanent first molars and deciduous second molars because in these researches, authors classified the teeth according to their diameter into normal, small, and large, so if the primary molar is large, the permanent molar will be large too and vice versa. On the other hand, Hussain et al. [18] found a strong, direct, and highly significant correlation between these teeth. In the present study, the teeth were taken collectively without segregation and the findings revealed a direct, moderate, and highly significant correlation. This comes in agreement with the findings of Gonna et al. [17] and partially with those of Hussain et al. [18]. Accordingly, regression equations were developed to predict the widths of permanent first molars from the widths of the deciduous second molars.

The actual and predicted widths were then compared using a paired *t*-test, and the results showed a nonsignificant difference; that is to say, the predicted width can be used to estimate the width of the actual permanent first molars, even before their eruption.

## 5. Conclusions

No significant side differences were found in the mesiodistal widths of teeth. However, a highly significant gender difference was found, except for that of the mandibular permanent first molar. The relationships between the teeth were found to be highly significant and led to the development of regression equations that can predict the widths of the permanent first molars from the widths of the deciduous second molars.

## Figures and Tables

**Table 1:** Descriptive statistics and side difference of the mesiodistal widths of the teeth.

Genders	Tooth	Descriptive statistics				Side difference		
		Right		Left				
		Mean	S.D.	Mean	S.D.	Mean difference	*t*-test	*p* value
Males	Maxillary 6	10.532	0.407	10.540	0.446	−0.008	−0.166	0.870
	Mandibular 6	10.648	0.430	10.632	0.444	0.016	0.485	0.632
	Maxillary E	9.180	0.443	9.128	0.422	0.052	1.915	0.067
	Mandibular E	9.756	0.473	9.796	0.462	−0.040	−1.333	0.195

Females	Maxillary 6	10.052	0.533	10.060	0.520	−0.008	−0.464	0.647
	Mandibular 6	10.588	0.379	10.568	0.396	0.020	2	0.057
	Maxillary E	8.760	0.465	8.744	0.465	0.016	0.848	0.405
	Mandibular E	9.508	0.467	9.532	0.445	−0.024	−1.186	0.247

**Table 2:** Descriptive statistics and gender difference of the mesiodistal widths of the teeth.

Tooth	Descriptive statistics				Gender difference		
	Males		Females				
	Mean	S.D.	Mean	S.D.	Mean difference	*t*-test	*p* value
Maxillary 6	10.536	0.423	10.056	0.521	0.480	5.059	0.000
Mandibular 6	10.640	0.433	10.578	0.383	0.062	0.758	0.450
Maxillary E	9.154	0.429	8.752	0.460	0.402	4.521	0.000
Mandibular E	9.776	0.463	9.520	0.452	0.256	2.798	0.006

**Table 3:** Relation between the mesiodistal widths of the permanent first molars and primary second molars and regression equations for prediction.

Genders	Tooth	*r*	*p* value	Equation
Males	Maxillary 6	0.352	0.012	*Y* = 7.361 + 0.347*X*
	Mandibular 6	0.591	0.000	*Y* = 5.238 + 0.553*X*

Females	Maxillary 6	0.409	0.003	*Y* = 5.999 + 0.464*X*
	Mandibular 6	0.312	0.027	*Y* = 8.053 + 0.265*X*

*Y* = mesiodistal widths of the permanent first molars, *X* = mesiodistal widths of the primary second molars.

**Table 4:** Descriptive statistics and method difference of the mesiodistal widths of the teeth.

Genders	Tooth	Descriptive statistics				Method difference		
		Actual		Predicted				
		Mean	S.D.	Mean	S.D.	Mean difference	*t*-test	*p* value
Males	Maxillary 6	10.536	0.423	10.537	0.149	−0.001	−0.026	0.980
	Mandibular 6	10.640	0.433	10.644	0.256	−0.004	−0.084	0.934

Females	Maxillary 6	10.056	0.521	10.060	0.213	−0.004	−0.058	0.954
	Mandibular 6	10.578	0.383	10.576	0.120	0.002	0.043	0.966

## Data Availability

The data used to support the findings of this study are available from the corresponding author upon request.
